# Current challenges and pitfalls in the pharmacological treatment of depression

**Published:** 2015

**Authors:** O Popa-Velea, IR Gheorghe, CI Truţescu, VL Purcărea

**Affiliations:** * Department of Medical Psychology, “Carol Davila” University of Medicine and Pharmacy, Bucharest; ** ”Carol Davila” University of Medicine and Pharmacy, Bucharest; *** Department of Marketing, “Carol Davila” University of Medicine and Pharmacy, Bucharest

**Keywords:** depression, medication, therapy

## Abstract

The multifactorial etiology of depression obliges needs an individual assessment, the psychopharmacological approach involving a biopsychosocial analysis for each individual case.

The rebalancing of the depressive patient, seen as a return to a normal level of psychosocial functioning and reduced risk of relapse is achieved with a prompt and constant support of specialized teams. Treatment should include psychopharmacological and psychosocial approaches, the results being interrelated and contributing to the prognosis of the disorder.

Progress in clinical and pharmacological research, vivid dynamics of socio-economic environment, the complexity of diagnostic evaluation and the need for an interdisciplinary approach may cause difficulties in addressing the depressive patient and the ethical controversies. The aim of this paper is to present a brief analysis of challenges encountered in the present psychiatric practice, starting from the heterogeneity of depressive manifestations and finishing with the prioritization of interventional forms.

## Introduction

In the last five decades, one of the strong tendencies in psychiatry and psychopharmacology has been towards an exponential increase of the number of drugs available for the treatment of mental disorders. Within this rich landscape, anti-depressive medication has a leading position, as depression remains one of the most critical problems of public health [**1**], with a lifetime incidence of 20-35% [**2**] and important costs for the individual and the society [**3**]. However, as important as it may seem, initiating or continuing the anti-depressive treatment can represent a genuine challenge for normal medical practitioners and can hinder a number of pitfalls.

**Lack of homogeneity of depression itself**

Depressed patients should be subdivided, according to many authors [**4**-**7**] based on their clinical features, response to treatment, and several measures of physiological function. The most commonly used distinction is between the “endogenous” depression (with more severe clinical features, likely to respond well to anti-depressant drugs, often inheritable, and potentially caused by biological abnormalities) and the “neurotic” or “reactive” depression (with opposite characteristics, including a significant response either to drugs or placebo). This classification is important, not only for the physician, in taking the decision of prescribing or not prescribing anti-depressive drugs, but also in assessing the apparent “inconclusive therapeutic results” obtained in heterogeneous samples of patients, comprising both kinds of depression. 

In terms of the comparative contribution of the genetic or environmental factors to the onset of depression, the first account averagely for only 16% of the variance in total depression scores [**8**], with even lower percentages when referring to self-reported symptoms. This explains in part the frequent reluctance of using anti-depressive medication in common practice, as these drugs often seem exaggerated or even inappropriate.

**Mechanisms of action, not always conspicuous**

All the currently available anti-depressants are known to alter the function of neurotransmitters in the central nervous system, such as serotonin, dopamine or norepinephrine. Yet, explaining the anti-depressant efficacy only by a direct effect on neurotransmitters is likely to be incorrect, as this cannot elucidate the therapeutic lag between the relatively immediate effects of anti-depressants in increasing the levels of the available monoamines in the synapse and the several weeks required to note the clinical alleviation of depressive symptoms [**9**]. Therefore, current research on understanding the therapeutic action has shifted away from the acute effects of anti-depressants in the synapse itself to the chronic membrane changes and adaptations within the cell that may take place following the prolonged exposure to these agents [**10**]. For example, in the last few years, the effects beyond the cell surface receptor sites have been more substantially studied, with an emphasis on the role of the intracellular second messenger protein systems and their effects on intracellular protein synthesis. 

**Methodological flaws**

The literature in the field is populated by a vast amount of studies that lack the comparative double-blind treatment vs. placebo vs. baseline treatment paradigm. Greenberg and Fisher did an extensive analysis of the strengths and deficiencies of the data on which the various reviewers based their conclusions [**11**]. It was reported that the degree of blindness is crucial in such studies and that the blinder the study participants are subjected to, who is getting the active drug and who is receiving the placebo, the more modest the advantage for anti-depressants becomes [**12**,**13**]. Moreover, the problem becomes more compelling with the discovery that patients and personnel involved in blind drug testing are afforded numerous cues (through such factors as body sensations and side effects) that help them distinguish between drugs and placebos [**14**-**16**]. Generally, numerous studies showed that the clinician’s ratings of anti-depressant outcome also tend to be more liberal than the patient ratings and are more likely subject to bias [**17**-**19**]. Unfortunately, the alternative use of solely the patient’s ratings is also a problem, given the insight problems most depressive patients face. 

**Controversies pertaining to the real efficacy of a given category of drugs**

SSRI (serotonin selective reuptake inhibitors) are a typical example of drugs that have earned a genuine popularity along time, currently making them the most extensively used anti-depressant agents. This position mainly stems from their relatively benign side effect profile, their ease of administration (once daily dosing for all agents), and the fact that their initial dose is close to the therapeutic dose, thereby making careful slow-dose titration unnecessary.

In spite of these genuine advantages, all SSRIs have the potential of side effects, which can fade over time (nausea, activation) or, on the contrary, tend to remain stable (sedation, sexual side effects). Moreover, the ambivalent effect of SSRIs, at least on parameters such as mood [e.g. sedation (paroxetine, fluvoxamine) vs. activation (fluoxetine, sertraline)] make some patients skeptical about their true effect. This can be in turn fed further by their capacity to alter the metabolism of other medications through the P450 system, a group of hepatic enzymes that metabolize foods, toxins and medications [**2**,**7**].

**The risk-benefit ratio**

Despite the progresses made by the pharmacological industry in the last decades, currently there is no anti-depressive drug that can be considered tailored to most patients who require anti-depressive medication. For some patients, the benefits greatly exceed the risks (exposing them to drug abuse, with consequences such as tolerance and psychological addiction), whereas other patients may become non-compliant (even if they get a unique moderate side effect, this is perceived as significant).

 Bupropion, for example, is an agent with effects on norepinephrine and dopamine and no serotoninergic effects. It is a stimulating anti-depressant and generally has among its side effects, insomnia, anxiety, tremor and headache. It has certain advantages that can be described as significant, especially in younger patients (e.g. very few sexual side effects, no weight gain). However, for the very same age category, there is equally a reason of worry, given the bupropion’ propensity to cause seizures and it is also contraindicated in patients with active eating disorders, such as bulimia nervosa or anorexia nervosa (they lower the threshold for seizures, via electrolyte abnormalities) [**7**,**20**].

Venlafaxine, a second example, may show a greater efficacy than the SSRIs, especially for more severely depressed individuals. However, as venlafaxine doses increase, a dose-related hypertension may emerge, which can affect a significant proportion (up to 9%) of treated patients at high dose [**21**]. 

A good example of the positive use of side effects is provided by Mirtazapine. This drug has a complex mechanism of anti-depressant activity, including presynaptic noradrenergic blocking (thereby enhancing noradrenergic function) and secondary enhancement of serotoninergic activity. The dosing is relatively simple, but side effects, such as sedation and weight gain, are common. However, these effects can make mirtazapine ironically helpful in those depressed patients who associate anorexia, agitation and insomnia [**7**,**22**].

The risk-benefit ratio for a certain drug can also be a matter of learning from history. Initially, tricyclic anti-depressives were seen as very potent, having a low cost, allowing a convenient once-a-day dosing, suitable for depression associated with chronic pain, and are easily manageable. However, soon, it was found that tricyclics have some major disadvantages, including the potential toxicity, the need to gradually increase the dose to obtain the full effect [**23**,**24**]. The side effects eliminated them from the first-line of choice in treating depression, for most clinical situations.

**Types and severity of the depression**

The psychotic depression (DSM-IV-TR’s “major depression with psychotic features”, characterized by mood congruent or mood incongruent delusions and hallucinations, added to the depressive background) [**25**] responds less well to anti-depressants alone than to anti-depressants plus an antipsychotic or electroconvulsive therapy. The atypical depression (DSM-IV-TR’s “depression with atypical features”, characterized by mood reactivity, and two or more of the following: significant weight gain or increase in appetite, hypersomnia, leaden paralysis and long-standing pattern of interpersonal rejection sensitivity) responds preferentially to MAO inhibitors, but given the improved side effect profile, SSRIs are typically prescribed first. Rejection sensitivity is especially very reactive to SSRI.

Severe depression typically responds better to non-SSRIs than to SSRIs. In studies that measured hospitalization duration, Hamilton depression scores (usually over 25) or occurrence/ persistence of melancholic subtype symptoms, tricyclics, mirtazapine and venlafaxine had a typically better efficacy than other anti-depressives [**26**].

The *perceived* severity of depression can influence the self-rated efficacy of the treatment. In general practice, most patients have mild to moderate depression and are probably less tolerant to adverse drug reactions. In contrast, in severely depressed patients, the lack of effect is more important than the development of adverse effects. The discontinuation rates due to adverse effects are generally higher in subjects treated with tricyclic anti-depressants than in subjects treated with selective serotonin reuptake inhibitors. 

**Somatic comorbidity**

In fact, the co-occurrence of a somatic disease can contribute to the etiology of depression, so an anti-depressive treatment, without a treatment of the somatic disease, is unlikely to show any effect on a long term. On another hand, a somatic disease can impede the administration of certain anti-depressive drugs, especially if sensitive organs for their pharmacokinetics (like liver or kidney) are involved. Moreover, some drugs can have direct adverse effects at the somatic level, thereby aggravating a previous condition or even creating a life danger (e.g. venlafaxine or MAO in hypertension patients).

In comorbid depression, the blood level of the drug can be a useful criterion to monitor, especially for drugs like nortriptyline, amitriptyline, clomipramine, and imipramine, that have fairly good relations between concentration and effect [**7**,**9**,**27**]. Therapeutic drug monitoring may be helpful in establishing the optimum dose of these drugs and for investigating treatment failure, especially since anti-depressive drugs are often subject to non-compliance. Monitoring can be important also because many non-compliant depressive patients do not seek help for this purpose.

**Switches of the anti-depressant**

Switching to another anti-depressant generally brings the dilemma of switching within-class or between-class of anti-depressants. Generally, switching to a drug with an identical mechanism was found effective in some cases, but there are very few studies that compare switching within the class to switching to anti-depressants from another class, so, overall, the present data are not conclusive [**11**,**28**]. In case of switching, it may be necessary to have a drug free interval before starting the new treatment to avoid drug interactions. 

**The balance pharmacology-psychotherapy**

The role of the psychologists in approaching depression has increased steadily in the last decades, as they have to perform new and critical therapeutic roles, such as: (a) to be the first to identify depression and to have to refer distressed individuals to physicians for medication treatment; (b) to be asked by physicians for input regarding diagnostic and medication issues; (c) to pursue and obtain prescription privileges; and (d) to be pressured by managed care programs to refer clients for medication evaluations. This new distribution of roles lies in a sharp contrast to the traditional representation of depression as a “medical illness” [**29**], with psychiatrists being the only specialists who have the ability to diagnose and address it. In fact, psychotherapeutic treatments often have a remarkable efficiency in alleviating symptoms, but also in improving psychosocial functions of the patient. They can also deal with the long-lasting effects of sudden exposure to intense stressful circumstances, where the benefit of pharmacological treatment is hardly else than symptomatic. 

Generally, the association of psychotherapy and pharmacological treatment has not been evaluated as producing dramatic increases over the effects achieved with drug therapy alone or psychotherapy alone. However, its use appears helpful and appropriate when the response to the initial course of treatment of several months’ duration is unsatisfactory, when symptoms recur, or when longer-range outcomes and the lengthening of the interval between episodes of relapse or recurrence are the focus of attention.

Cognitive-behavioral therapy is a form of psychotherapy that proved to reduce the risk of relapse after the termination of the pharmacological treatment [**30**]. In addition, the outcome of psychotherapy appeared to be roughly comparable to medication in the treatment of the acute episode; this is remarkable, given the cost-effectiveness of psychotherapy, generally higher compared to medication. Several meta-analyses—reported in both psychiatry and psychology journals—covering multiple studies with thousands of patients are consistent in support of the perspective that psychotherapy is at least as effective as medication in the treatment of depression [**31**]. Psychotherapy is effective for both vegetative and social adjustment symptoms, especially when the outcome is assessed with patient-rated measures and when long-term follow-up is considered. It can teach skills to help prevent depression, making such treatment an attractive, cost-effective alternative to drug treatments.

**Fig. 1:**
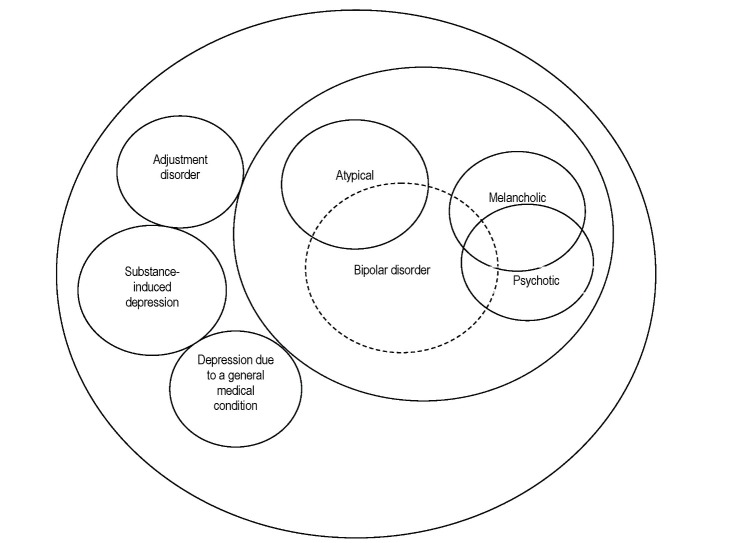
Heterogeneity of depression

**The risk/ benefit ratio of a high accessibility to medical information**

Among the factors that could influence the way depression treatment is seen by patients and their relatives, the accessibility to medical information is seen by many clinicians as a key one. The need of explaining one’s symptoms, but also the need of knowing more about the profile of a certain drug which is recommended by the physician or simply looks more potent, leads to what is called in current literature “health information seeking behaviour” (HISB) [**32**]. HISB is a strategy that can be adopted by certain individuals, including depressive patients, in order to better cope with threat, via an increase in their ability to evaluate the situation. Generally, it does not include the cases in which the person is unwillingly exposed to medical information, or is passive, or is simply remembering without effort a certain medical detail (although some authors, such as Case, advocate for a larger definition of HISB). Another circumstance in which HISB cannot be invoked is the situation in which the patient obtains certain information from the physician, without asking it explicitly [**33**].

Seeking for medical information in depression is commonly met, especially when depression is in its incipient phase. It can help patients in diminishing their ignorance and confusion and can encourage them make more appropriate decisions, including medical ones. Online web resources are especially welcomed, as medical information is organized in an easy way and is readily available. Some web resources are subject of high sharing on popular social network, thereby gaining a sometimes-undeserved popularity. This phenomenon is mediated by the personal touch of the shared experience, where for example a certain drug proved to be quite close (or on the contrary, quite far away) from the social norms or influences commonly accepted by a group or a community. This in turn can influence the recruitment rate of new drug consumers and also their understanding of the disease and the treatment.

Unfortunately, the easy access to internet resources has its pitfalls, among the highest being the decrease of access to the experts in the domain. Especially in the delicate field of mental illness, many patients can see the recourse to the consultation from the part of a specialist as an insurmountable difficulty. Instead, finding information, diagnosis criteria and also psychotropic medication is rather easy in the online environment, this leading to a substantial risk of abuse and inappropriateness in administrating the required medication for depression. The decision of taking/ interrupting a certain drug is consequently determined in a large proportion by lay opinions, such as those stemming from friends or former/ current patients. The phenomenon is self-fueled, as health services are archetypical according to their nature, therefore characterized by a substantial body of shared lay knowledge.

The virtual communities created in relation to health, in particular to solving most common mental issues are quite strong and increasingly popular, irrespective of the country and of the demographic profile of their users. In a majority of cases, these virtual communities have a positive role, as they offer alternatives of diagnosis and treatment, support and counselling from those members who have faced the same problem. However, in such communities it is often the case to get to a common denominator of simplistic beliefs about a certain disease or a certain medication, this set of beliefs being later reinforced and spread with a redoubtable efficacy via classic websites, but also via social networks, chat rooms and forums. These phenomena create the basis for a veritable electronic word-of-mouth marketing (eWOM) in an economical and social sense [**34**].

To obtain a better approach of depressive patients, the existence of HISB and eWOM should be taken into account, especially in what concerns their function and their use in constructing a better management of health services [**35**,**36**].

## Conclusions

Although medication is often a matter of therapeutic protocol, in treating such a multifaceted disease as depression, the clinician should consider all the factors potentially impairing the pharmacological effect and take decisions accordingly. The use of older drugs is equally legitimate, if they fit better in the clinical context of the patient. Altogether, in the treatment of depression, there are not new and old drugs, instead we should think about using molecules addressing different needs. The access to medical information and their influence on patients should be considered. As a whole, a careful clinical assessment of each case should not be substituted by generic protocols and should be used regularly as a necessary and powerful approach in addressing every depressed patient.

**Sources of funding**

This article was funded from personal sources.

**Disclosures**

None.
